# Adult patient and caregiver perspectives on the impact of NF1-PN: Insights from a US qualitative survey

**DOI:** 10.1093/noajnl/vdag033

**Published:** 2026-02-16

**Authors:** Phioanh L Nghiemphu, Conrad L Cordova, Alyssa Bowling, Abby Crites, Xiaoqin Yang, Theresa Dettling

**Affiliations:** Department of Neurology, David Geffen School of Medicine, University of California, Los Angeles (P.L.N.); Patient author (C.L.C.); Global Medical Affairs, Alexion, AstraZeneca Rare Disease, Boston (A.B.); Advisory & Brand Intelligence, IQVIA, Durham (A.C.); Value and Implementation Outcomes Research, Merck & Co., Inc, Rahway (X.Y.); US Health Economics and Outcomes Research, Alexion, AstraZeneca Rare Disease, Boston (T.D.)

**Keywords:** adults, caregiver perspectives, neurofibromatosis type 1, patient perspectives, plexiform neurofibroma

## Abstract

**Background:**

Adults with neurofibromatosis type 1 and plexiform neurofibroma (NF1-PN) experience multiple clinical symptoms and diverse manifestations, which place a burden on patients and impact quality of life. This qualitative study aimed to improve understanding of disease burden, healthcare experience, and unmet needs of adults with NF1-PN in the United States, from patient and caregiver perspectives.

**Methods:**

Telephone interviews were conducted with adults who had a diagnosis of NF1-PN, or with caregivers, focusing on patient background, diagnosis, interactions with healthcare professionals, disease and symptom management, transition of care, and unmet needs.

**Results:**

The study included 11 adult patients with NF1-PN and 2 caregivers; 85% of patients were diagnosed in childhood. Patients lived with multiple conditions associated with NF1, including pain disorders, psychiatric disorders, and chronic migraines. NF1-PN impacted daily living, work, school, relationships, and mental and emotional health. Most patients (82%) transitioned from pediatric to adult care, although there was variability in the transition experience. Some dropped out of care (23%) due to various factors, including time constraints, physician location, financial insecurity, lack of insurance, and perception of no available treatments/cure. Medical management primarily comprised medications to relieve symptoms associated with NF1 manifestations. Respondents identified a need to be more informed about their care. Improved treatment options for NF1-PN are desired, particularly medications that stop or slow PN growth.

**Conclusions:**

The study demonstrated that NF1-PN has a profound impact on adult patients’ lives. Several unmet needs exist for the adult population, including medications to treat the PN and its associated symptoms.

Key Points

- Patients have many NF1 manifestations, including pain and psychiatric disorders.
- NF1-PN impacts daily life, work, school, relationships, and mental/emotional health.
- Adults want to be more informed about and have better treatment options for NF1-PN.

Importance of the Study

This qualitative study aimed to improve understanding of disease burden, healthcare experience, and the unmet needs of adults with neurofibromatosis type 1 and plexiform neurofibroma (NF1-PN) in the United States, based on interviews with patients and caregivers. Patients reported living with multiple conditions associated with NF1, including pain disorders, psychiatric disorders, and chronic migraines. NF1-PN had a profound impact on daily living, work, school, relationships, and mental and emotional health. Some patients reported dropping out of care as they transitioned from pediatric to adult settings, due to factors such as time constraints, physician location, financial insecurity, lack of insurance, and perception of no available treatments/cures. Key unmet needs included a desire to be more informed about NF1-PN and improved NF1-PN treatment options, including medications that slow or stop PN growth. These findings provide a unique patient and caregiver perspective on the burdens experienced and highlight unmet needs in this patient population.

Neurofibromatosis type 1 (NF1) is a genetic condition caused by mutations in the *NF1* tumor suppressor gene, with an estimated prevalence of approximately 1 in 3000 to 1 in 4000 people, depending on country and region.^1–4^ The clinical manifestations of NF1 are diverse, and may involve multiple organ systems, which requires patients to seek care from a wide range of medical and surgical specialists.^3^^,^^5^^,^^6^ Manifestations may include plexiform neurofibroma (PN), cognitive dysfunction, skeletal abnormalities, neurologic symptoms, cutaneous neurofibroma, and malignant peripheral nerve sheath tumors.^6^^,^^7^ Adults with NF1 also report pain at a higher frequency than pediatric patients with NF1, and typically report multiple sources of pain, including PN, migraines, cutaneous neurofibroma, pruritus, scoliosis or bone dysplasia, increased pressure on nerves, and malignancies.^8^

Up to 50% of individuals with NF1 develop PN. PN may be associated with significant clinical symptoms, including pain, disfigurement, motor dysfunction, airway dysfunction, visual impairment, and bladder/bowel dysfunction.^5^^,^^6^^,^^9–13^ Although PN tend to grow more rapidly in childhood, they may continue to grow at a slower rate in adults.^5^^,^^6^^,^^9–13^

The presence of PN and its associated complications may place a significant burden on patients, reducing their ability to perform daily activities and impacting their quality of life (QoL).^11^ In adults, PN can cause self-consciousness of appearance as well as the inability to form or maintain relationships, achieve independence, fulfill family and work roles, and experience pleasure.^14^^,^^15^

Inequalities in NF1 management between pediatric and adult patients have been identified and transition of care into adulthood remains challenging.^16–19^ There are fewer specialty clinics dedicated to adult patients as compared to pediatric patients, and many adults with NF1 are referred back to their general practitioner.^7^^,^^18^^,^^20^ Patient-related factors can also create barriers to successful healthcare transition, including unawareness of where to seek care, a belief that nothing can be done to manage their PN, limited decision-making abilities, stress, and financial concerns.^17^^,^^20^^,^^21^

Current management recommendations for adults with NF1 reflect the diversity of manifestations and their extensive impact, as well as the need for ongoing lifelong medical care and health maintenance, including monitoring for malignancies, pain, and mood disorders;^7^^,^^22^ family planning, contraception, genetic counseling, and high-risk obstetric care;^7^^,^^23^ breast cancer screening for women aged ≥30 years;^7^^,^^22^ monitoring for osteoporosis and scoliosis;^6^^,^^7^ and vitamin D supplementation.^6^^,^^7^ However, there are limited management options recommended or available that are specifically aimed at treating PN, despite their prevalence in individuals with NF1.^6^^,^^24^^,^^25^ Selumetinib (ARRY-142886, AZD6244), a potent, selective oral inhibitor of mitogen-activated protein kinase kinases 1 and 2 (MEK1/2), was first approved in the United States in 2020 for pediatric patients (aged ≥2 years) with NF1 and symptomatic, inoperable PN. Additionally, in September 2025, the US FDA extended the approval of selumetinib to pediatric patients aged ≥1 year with symptomatic, inoperable PN. This approval was based on exposure matching between pediatric patients in the SPRINT capsule formulation study (aged ≥2 years) and the SPRINKLE oral granule formulation study (aged ≥1 year).^26^ FDA approval of selumetinib for the treatment of adults with NF1 and symptomatic, inoperable PN was granted in November 2025, and was based on the results of the international, randomized, double-blind, placebo-controlled, Phase 3 KOMET study.^27–29^ KOMET demonstrated that selumetinib treatment leads to PN reduction and clinically meaningful reduction in PN-related pain intensity in adults with NF1 and symptomatic, inoperable PN.^30^ In February 2025, mirdametinib was FDA approved in the United States for adult and pediatric patients (aged ≥2 years) with NF1 and symptomatic PN not amenable to complete resection.^31^ No approved treatments for adult patients with NF1-PN were available throughout the time of data collection or during the conduct of this study.^30^

This qualitative study aimed to improve understanding of the disease burden, healthcare and treatment experiences, and unmet needs of adults with NF1-PN in the United States, from the perspective of patients and caregivers. The study included both adult patients as well as caregivers of adult patients to identify and understand any differences in perspectives and challenges between these groups.

## Methods

### Design and Participants

Caregivers and adults with NF1-PN were recruited via email using the IQVIA Rare Disease Patient Panel. To identify and recruit participants, a dedicated IQVIA outreach team contacted HCPs and patient support groups via email, and utilized social media platforms. No patient information was shared by HCPs. The study took place between July 14, 2023, and September 6, 2023, and data were analyzed from 45-min, double-blind telephone interviews conducted in the United States between July 27, 2023, and August 4, 2023. Eligible participants declared a diagnosis of NF1-PN or were caregivers of adult patients with NF1-PN. All participants were required to be involved in NF1-PN treatment decisions, to have no conflicts for inclusion (eg, due to employment or state law) and to be ≥18 years of age. Caregivers were non-NF1 specialists and were required to be currently assisting or providing care for an adult with NF1-PN. Respondents who did not meet these criteria were screened out and did not proceed with the survey. Honoraria were provided at fair market value for 45-min interviews. Due to the rarity of NF1, a saturation threshold approach was not used; however, 12-20 participants were deemed sufficient to provide directional insights to identify themes and answer research questions.^32^

To provide a broad picture of the patient and caregiver experience, a diverse participant population was recruited. Participants were categorized after interviews had been completed into the following subgroups for comparison: younger (aged 18-34 years) versus older participants (aged ≥35 years), to understand differences in burden across the lifespan of adults with NF1-PN; patients who had transitioned to an adult care team versus those who had remained with their pediatric care team; and those who were diagnosed as adults versus those who were diagnosed as children. Information on treatment setting (eg, Center of Excellence or hospital), insurance type, and region were collected during recruitment with efforts made to include a diverse set of participants.

All participants were interviewed individually, and caregivers who participated were not associated with any of the patients who participated. All interviews were facilitated by the same moderator. Moderator bias was limited by the use of discussion guides, which contained a set of questions covering patient background and NF1-PN diagnosis, level of HCP engagement (such as physician specialty and appointment frequency), transition of care, NF1-PN pain management, and unmet needs. A copy of the discussion guides for patients with NF1-PN and caregivers of patients with NF1-PN are included in the Supplementary Methods S1 and S2.

The interviews were designed to obtain qualitative data on patient and HCP relationships, symptoms, disease progression, disease management, unmet needs in the treatment of adult patients with NF1-PN, and impact of insurance coverage and treatment costs.

During the interview, participants were asked to rate the impact of NF1-PN using a 5-point scale (1 = not at all impactful; 5 = extremely impactful) on the following parameters: ability to participate in school/work, personal relationships with family and friends, mental health, and overall quality of life. Verbatim transcripts were developed from the audio recordings of interviews; any personally identifiable data were removed, and transcription errors were corrected. Thematic analysis of the data was performed, in which key themes were identified and coded by a qualitative data analyst, and guided by a flexible coding approach.

### Analyses

Descriptive analyses were used to describe interview findings, including means for continuous variables, and proportions and frequencies for categorical variables. The study was intended to be exploratory in nature, and no statistical analyses were undertaken.

### Funding and Data Privacy

Data were collected, analyzed, and stored in accordance with standards established by the Council of American Survey Research Organizations. Participants were informed that responses to surveys in this study were de-identified prior to analysis and publication. Alexion, AstraZeneca Rare Disease was responsible for the development of the patient/caregiver questionnaires, interviews, and data analysis. Alexion, AstraZeneca Rare Disease funded the study and medical writing assistance and provided formal review of the publication. The authors retained control and final authority of publication content and decisions, including journal choice.

## Results

### Demographics

In total, 42 patients and caregivers completed screening. Of those who completed screening, 13 (31%) were eligible for inclusion in the study; this included 11 adult patients with NF1-PN and 2 caregivers of adult patients with NF1-PN. Caregivers answered demographic and diagnostic questions about the person they care for.

Patients were predominantly aged ≥35 years (62%; 8/13) with a mean (range) age of 37 years (24-49); 92% (12/13) were female and 8% (1/13) were male. Most patients (85%; 11/13) had been diagnosed with NF1-PN in childhood; of those, 82% (9/11) had been transitioned to settings for management of adults with NF1-PN. Patients varied in the level of their knowledge of NF1-PN, with 27% (3/11) reporting they considered themselves extremely knowledgeable, 36% (4/11) very knowledgeable, and 36% (4/11) somewhat knowledgeable. One caregiver (50%) reported that they considered themselves extremely knowledgeable and the other caregiver (50%) reported that they considered themselves very knowledgeable. Patient age at the time of the study, age at diagnosis, care status, and level of knowledge of NF1-PN are shown in Table 1.

**Table 1.** Demographics and disease characteristics at the time of the survey

Characteristic, *n* (%)	**Patients (*N *= 13)** ^a^
**Current age, years**	
18-34	5 (38)
≥35	8 (62)
**Region of United States**	
Midwest	4 (31)
Northeast	3 (23)
South	4 (31)
West	2 (15)
**Time of NF1-PN diagnosis**	
Childhood	11 (85)
Adulthood	2 (15)
**Level of knowledge of patients**	*n = *11
Extremely knowledgeable	3 (27)
Very knowledgeable	4 (36)
Somewhat knowledgeable	4 (36)
**Level of knowledge of caregivers**	*n = *2
Extremely knowledgeable	1 (50)
Very knowledgeable	1 (50)
Somewhat knowledgeable	0 (0)
**Care status of individuals diagnosed as children**	*n = *11
Transitioned to adult HCP	9 (82)
Still sees childhood HCP	1 (9)
Currently not seeing an HCP	1 (9)
**Current treatment setting**	
HCP’s office	6 (46)
Hospital setting	4 (31)
NF1 Center of Excellence	2 (15)
Not currently seeing HCP	1 (8)
No response provided	2 (15)
**Insurance type**	
Private (PPO/HMO)	6 (46)
Medicare	4 (31)
Medicaid	2 (15)
Not insured/self-pay	1 (8)

**Abbreviations**: HCP = healthcare professional; HMO = health maintenance organization; NF1 = neurofibromatosis type 1; PN = plexiform neurofibroma; PPO = preferred provider organization.

a The total sample size of 13 comprises 11 patients and 2 caregivers; caregivers answered demographic and diagnostic questions about the person they care for.

Almost half the patients (46%; 6/13) were being treated in the setting of a HCP’s office for NF1-PN, while 31% (4/13) were treated in a hospital setting; of these, 2 (15%) indicated that they were under the care of an NF1 Center of Excellence. Overall, 8% (1/13) of patients were not currently seeing a doctor for NF1-PN, and the remaining 15% (2/13) did not respond to this question. Patients’ locations were geographically spread across the United States. Nearly half had private insurance (46%; 6/13), while 31% (4/13) had Medicare, and 15% (2/13) had Medicaid; 1 patient (8%) reported that they had no insurance and were self-funding their care. Treatment setting, geographic regions of patients, and insurance type are shown in Table 1.

### NF1/NF1-PN Diagnosis

Of the patients diagnosed during childhood (85%; 11/13), 6/11 (55%) recalled that their pediatrician was the first to suspect a diagnosis of NF1-PN and that this was based on the extent and visibility of symptoms and the presence of PN at the time. In patients whose pediatricians initially suspected NF1-PN (55%; 6/11), the majority (67%; 4/6) were subsequently referred to other specialists or a team of specialists (such as geneticists [*n *= 4], neurologists [*n *= 1], oncologists [*n *= 1], neuro-oncologists [*n *= 1], neuro-ophthalmologists [*n *= 1], cardiologists [*n *= 1], or respiratory therapists [*n *= 1]) to confirm the diagnosis or manage care. In 2 cases, patients recalled that their pediatrician diagnosed and solely managed their care during childhood (18%; 2/11). Overall, 3/11 patients (27%) diagnosed during childhood mentioned that an NF1 specialist was involved in their diagnosis, with 2 mentioning neurologists and 1 mentioning a geneticist. The 2 patients diagnosed during adulthood recalled being diagnosed by a primary care physician or dermatologist during a routine visit.

### NF1-Associated Conditions

Study participants reported living with multiple other conditions that they associated with NF1; the most common of which were pain-related disorders (including fibromyalgia, neuropathy, and complex regional pain syndrome), psychiatric disorders (anxiety, depression, bipolar disorder, impulse control disorder, and attention-deficit hyperactivity disorder [ADHD]), and chronic migraines (Table 2). Three patients experienced brain tumors (astrocytoma) that they associated with NF1, while 1 patient reported a recent diagnosis of bone cancer but did not attribute it to NF1.

**Table 2.** Conditions experienced by patients with NF1-PN that patients and caregivers specifically associate with NF1

Condition	Number of participants
Pain disorders such as fibromyalgia, neuropathy, and CRPS^a^	10
Psychiatric disorders such as anxiety, depression, bipolar disorder, impulse disorder, and ADHD	7
Chronic migraines	6
GI issues such as GERD, gastroparesis, and gastroenteritis	4
Scoliosis	3
Brain tumors (astrocytomas)	3
Hearing loss, auditory processing issues	3
Vision issues	3
Epilepsy (as a result of brain surgery)	3
Memory loss	2

**Abbreviations**: ADHD = attention-deficit hyperactivity disorder; CRPS = complex regional pain syndrome; GERD = gastroesophageal reflux disease; GI = gastrointestinal; NF1 = neurofibromatosis type 1; PN = plexiform neurofibroma.

a Only 1 patient specifically mentioned CRPS; the majority of patients did not use this term.

### Impact of NF1-PN

**Figure 1.**
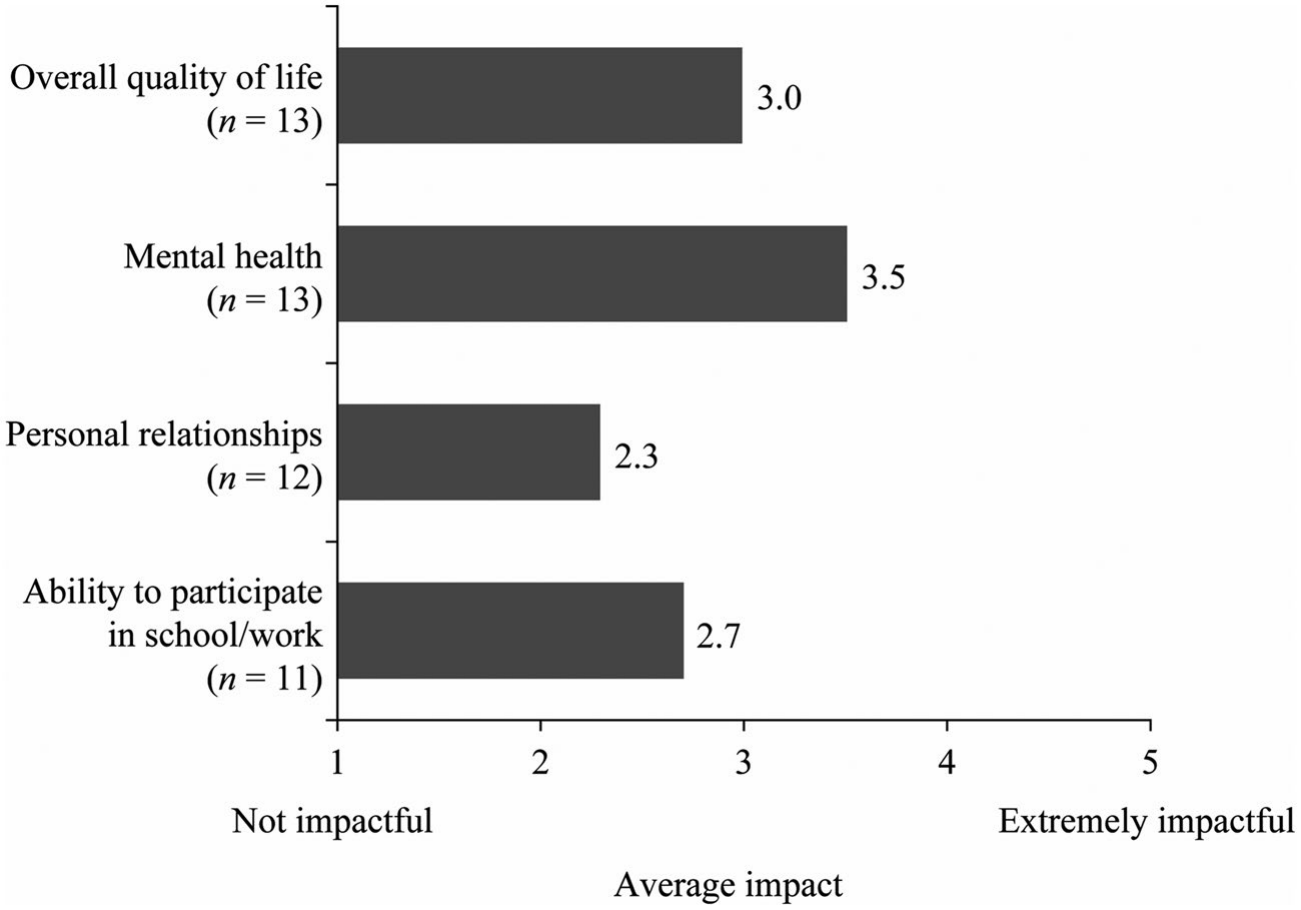
Impact of NF1-PN on patients’ lives on a scale of 1 to 5. Graphical representation of the average impact of NF1-PN on 4 aspects of patients’ life–overall quality of life, mental health, personal relationships, and ability to participate in school or work. A score of 5 is considered extremely impactful and a score of 1 is considered not impactful.

The impact that NF1-PN has on participants’ lives was found to be profound and to affect day-to-day living, work, school, relationships, mental health, and emotional health (Figure 1). While different symptoms and degrees of severity were described, all participants recognized the impact of NF1-PN on lifestyle. Of the 12 participants who rated the impact that NF1-PN had on lifestyle, 3 (25%) reported that it had a high impact and cited reasons such as chronic pain related to nerve sensitivity and tumor growth, chronic headaches, weakness, mobility issues or paralysis leading to the requirement for mobility aids and an inability to drive, low vision/vision impairment, low hearing/hearing impairments requiring hearing aids, and the requirement for psychiatric involvement to address mental health concerns. Four participants reported that NF1-PN had a moderate impact on lifestyle due to reasons such as cosmetic appearance/tumor visibility, the potential for requiring accommodations at school (eg, reader/note-taker) or work (eg, sitting close to the bathroom or exit, having a handicapped accessible desk), the need to be mindful of activities (eg, limitations with playing sports), and the requirement for psychiatric involvement to address mental health concerns. Four participants reported that NF1-PN had a low impact on lifestyle due to reasons such as cosmetic appearance/tumor visibility, or more cosmetic symptoms than pain; these participants described “just living with it.” One participant noted that NF1-PN had not affected lifestyle that much. Patients and caregivers described that differences in the patient experience may be related to the distribution and extent of PN/tumors (eg, prevalence, location, and visibility of tumors), the cosmetic impact of birthmarks and café-au-lait spots (eg, related to the distribution and visibility of spots), the severity of associated symptoms, or the extent of pain and associated disabilities.

### Frequency of Medical Care

Most participants (*n *= 10/13) noted that HCPs were visited on an annual basis, if not more frequently. Participants reporting regular medical care noted routine visits every 6-12 months with the primary neurologist or neuro-oncologist, and most also noted that ophthalmologists were routinely seen to monitor vision changes. These participants also noted that routine imaging, in the form of magnetic resonance imaging or computed axial tomography scans, are ordered every 6-12 months to monitor brain and spine changes for tumor growth, development of new tumors, or changes in nerve sensitivity. About half of the participants (*n *= 6/13) reported medical management by a multidisciplinary team, and for those participants, routine visits may be more comprehensive, covering the following: physical examination including visual field tests, walking tests, and reflex tests to determine changes in motor skills; discussions around any aesthetic changes to PN or the development of new PN; discussions around symptoms like headaches, increases in pain, or other bothersome symptoms; discussing general patient status and exploring problems, complaints, medications, and QoL issues, and discussions around any needs for surgery.

Several participants (*n *= 3/13) reported a lapse in healthcare at some point during adulthood, ranging from 1 to several years. The lapse in care was related to different factors, including scheduling constraints, location of HCPs (no NF1 clinic nearby), financial insecurity, and lack of healthcare insurance, as well as the perception of a lack of available treatments or cures for NF1-PN. For example, surgery was perceived to be cost-prohibitive and only a temporary, cosmetic solution. Some participants reported that care had been discontinued temporarily when there was dissatisfaction with the care team or physicians and a new or different care situation and/or different solutions to care were being sought.

### Medication Management

Medications used for PN symptom management include over-the-counter (OTC) therapies and prescription pain medications. Use of a variety of OTC medications were reported by participants, including non-prescription pain medications (*n = *9/13), skin creams/oils (*n *= 2/13), and vitamin supplements (*n *= 1/13). Prescription medications were used to address pain (*n *= 3/13; eg, pregabalin, oxycodone, ketorolac), headaches, anxiety/depression, blood pressure changes, sleep disorders, fibromyalgia, ADHD, and cancer (eg, chemotherapy). None of the respondents in this study reported receiving MEK inhibitors.

### Unmet Needs

When asked what could be changed about the way NF1-PN is managed, patients and caregivers identified an interest in being more informed about their care and disease state, and improved treatment options for NF1-PN as key aspects (Table 3).

**Table 3.** Patient and caregiver responses when asked what could be changed about the way NF1-PN is managed

Theme	Patient and caregiver responses	Number of participants	Relevant quotes
Better informed	More informed care and more disease state information	7	“I’d like more informed care or more access to newer research or newer options”
			“I’d like more general knowledge of it”
			“I have tumors on my optic nerves. I’m starting to have vision problems… I really, really want to try and be able to get seen. And I just don’t think even if I were to be able to get insurance that would cover it, that I would be able to use it at the doctor that I know knows about it”
			“More focus and more drive to manage everything that’s going on. To figure out why things are developing in the way they are developing”
			“I’d like to know why I’m experiencing this pain. More specific information”
			“Are these causing an issue where this needs to be removed?”
			“When you’re finally getting tested it’s 2 times, 3 times its size”
			“I’d like to know more about future prognosis, what could possibly happen, so I could be better prepared”
Improved treatment options	Treatments that decrease the size, severity, and growth of PNs	2	“I would like a treatment to help find ways to decrease the size, severity, and growth of the PNs”
			“I’d like to get rid of all my tumors and just have them all removed”
	New treatments that are specifically indicated for adults	1	“I would like a treatment for adults”
	More effective pain management	1	“Reduction of pain would be most valuable for her. If she didn’t have that, I think she would be a lot happier”
Access to research and developments	Easier access to new research, clinical trials, and medications	2	“What could be more helpful is probably more open access to different clinical trials and different options”
			“Easier access to new NF1-PN medications that are in development”
			**[Has your doctor talked to you about clinical trials?]** “They haven’t”
Centralized and coordinated care	Access to a care/case manager to help manage care, provide guidance and support, and prioritize care for patients	2	“I would like to have a care manager to help me navigate, to help me say, ‘This is what you need to do next’”
			“I’d like help to keep it [PN] in check, rather than finally deciding to get things investigated at the last minute”
			“It takes too much time trying to set up appointments with these doctors”
Consideration of all aspects of care	Greater access to HCPs to discuss topics such as family planning and genetic counseling	2	“I’m wondering how it would affect children”
			“There was a concern of how it would affect them” **[children]**
			**[Regarding family planning]** “More accessible information. A lot of it is not easily digestible. There’s not a lot of information out there in lay terms”
	More time with physicians and being heard	1	“If more doctors would listen a little more. Sometimes it’s being heard that can make a huge difference”
	More discussion around the impact of mental health issues like anxiety and depression on patients with NF1-PN	1	“More support with having a rough time, depression or anxiety”

**Abbreviations**: HCP = healthcare professional; NF1 = neurofibromatosis type 1; PN = plexiform neurofibroma.

There was variability in participant approaches to obtaining information about NF1-PN. Of the 10 participants responding to this question, 2 were actively involved in seeking and receiving information on a routine basis, while the rest were more reactive, only seeking information when they had a question or concern. The key sources of information used were clinical websites such as PubMed and WebMD (*n *= 4/10), support groups (specified and unspecified: *n *= 4/10), Googling NF1-PN (*n *= 3/10), social media such as Instagram and Facebook (*n *= 2/10), HCPs and care centers (*n = *2/10), advocacy groups/websites (*n = *2/10), Technology, Entertainment, and Design (TED) talks and videos (*n = *1/10).

Patient and caregiver involvement with patient support programs and advocacy groups was also mixed, with *n = *3/8 respondents reporting involvement in advocacy groups. Those involved in these groups often had more severe or debilitating disease. Those who were not involved in such groups gave the following reasons: not aware of any (*n = *3/5), and do not feel like they could relate to others since the disease varies among patients (*n = *2/5). Some participants (*n = *2/8) expressed the need for more patient-focused support groups and stated that they were interested in “communities” or places to share information, receive support, and access information on new NF1 treatments and medications.

### Medication Needs

The patients and caregivers in this study noted that they are highly open to new medications for the treatment of NF1-PN (Table 4); in particular, participants expressed that medications that stop or slow the growth of PN would be highly desirable. Other desirable characteristics noted by participants were medications that reduce the size/severity of PN, improve pain management, have minimal side effects, and are covered by insurance. Participants were interested in new treatments with strong clinical trial data and feedback from real patient experiences. Treatments with indications for both pediatric and adult patients with NF1-PN were also of interest.

**Table 4.** Patient and caregiver responses when asked about desired attributes for medications

Theme	Patient and caregiver responses	Number of participants	Relevant quotes
Reduce the size/severity of PNs	Shrink the size of PNs or make the PNs disappear altogether; at a minimum, make PNs less noticeable	8	“I would say shrink the size of the tumors”
			“To disappear”
			“To reduce the tumors or prevent more from growing if that’s even possible”
			“Make the neurofibroma less noticeable”
			“The key thing would be size management. Honestly, if the medication could get rid of it completely that would be so amazing”
			“If they could come up with a medication that could shrink the tumors, that would be amazing”
			“Probably just get rid of the tumors” “I don’t like people staring at me with the tumors”^a^
			“If they could make the tumors disappear, that would be great”
Slow or stop tumor growth	Slow and/or prevent the spread of PNs	6	“Just be a way to prevent more from growing if that’s even possible”
			“Make it so they stop growing. New ones stop coming and the ones that I already have don’t change at all”
			“Just stop the growth as much as it could or slow it down at some point”
			“One that would help prevent the tumor from growing more, so just stall their growth”
			“I would want one that would not make my tumors get bigger and not make me get any more tumors”
			“If there’s a medication that could treat the bumps, I know it’s impossible to have medication for that, but so the bumps don’t get out of control; that would be my biggest thing”^a^
Improve pain management	Medications that numb the pain and/or control the itchiness from PNs; address skin sensitivity; “make my nerves less sensitive to pain”	5	“Numb the pain and not have such sensitive nerves”
			“Reduce or remove the pain”
			“Managing pain and the sensitivity of the plexiform [neuro]fibromas”
			“Help with the itching. Help to where I’m not wanting to claw my skin off”
			“Just a medication that could help control the nerve pain would be great. Especially the electric shock-type pain, that would be wonderful”
Covered by insurance	Access and good coverage by insurance	2	“For insurance to cover it”
			“Insurance is an issue. Because of income, I can’t afford to get all of them removed”
Minimal side effects	Medications with no or low side effects, especially cognitive side effects like feeling drowsy, loopy, or foggy	1	“If it could not affect my cognition and not make me feel drugged or loopy, that would be amazing”

Abbreviations: NF1 = Neurofibromatosis type 1; PN = plexiform neurofibroma.

a While participants were asked about symptoms caused by/related to PN, it is possible that they may not have known whether signs and symptoms, such as chronic headaches or vision impairment, were PN related or were manifestations of NF1 that were independent of their PN.

### Transition of Care

Among patients who were diagnosed with NF1-PN as a child (*n = *11/13), 82% (9/11) had transitioned out of the care that they were receiving as pediatric patients to adult HCPs or care teams who manage NF1-PN. For 9 patients who had transitioned, the majority of respondents said that the transition occurred around the ages of 18-19 years. One patient reported that they did not transition until the age of 29 years. Insurance coverage was cited as a reason for transitioning care.

Transitioning out of pediatric care was typically initiated by the pediatric care team versus the patient. Five participants mentioned that they would have preferred to continue with the care given by pediatric HCPs versus moving to the adult care team but that they were outside of the pediatrician’s “care zone.” Pediatric care teams typically made referrals (*n = *6) to adult HCPs; however, in 3 cases, participants had to explore new care options on their own. There was variability in participants’ experiences with the transition process. Five described the transition as easy and/or uncomplicated, noting it was “easy” and “straightforward” (Supplementary Table 1). By contrast, others experienced challenges such as finding the right HCP to manage and treat NF1-PN (*n = *3), feeling that they were not being heard by the adult care team compared with the pediatric care team (*n = *2), and frustration with a different level or lack of care and understanding about NF1-PN symptoms (*n = *1).

## Discussion

In this study, adult patients and caregivers described their perspectives on the diagnosis and healthcare experience of NF1-PN, as well as unmet needs across different aspects of care. At the time of this study, no MEK inhibitors had been approved for use in the adult population. Previously, Copley-Merriman et al. (2021) described how no 2 patients are alike,^11^ with every patient having a different degree and severity of symptoms. As patient experiences can be very different, there is a need for individualized care. This variability was reflected in the results of this study, which showed that, despite patients and caregivers agreeing on several aspects of the disease and the impact on their life, there were substantial differences with regard to specific factors. The most common conditions related to NF1 included pain disorders, reported by more than 75% of respondents, and psychiatric disorders such as anxiety and depression, described by over half of the study population. However, multiple other comorbidities were reported, affecting between 2 and 6 individuals and ranging from memory loss to gastrointestinal issues and chronic migraines.

Participants reported differences in experiences with NF1-PN-associated symptoms. These differences related to the distribution and extent of PN/tumors, the cosmetic impact of birthmarks and café-au-lait spots on the skin, the severity of associated symptoms, being diagnosed with comorbid conditions, and the extent of disabilities. Participant reflections suggested that worsening of symptoms, more sensitivity, and increases in pain typically lead to a diagnosis of NF1-PN. Consistent with previous research,^11^^,^^33^^,^^34^ this study indicated that the impact of NF1-PN on patients’ lives can be profound, and for all patients, it had some level of impact on day-to-day living, work, school, relationships, and mental/emotional health. Participants who said that NF1-PN had a high impact on their lifestyle reported experiencing serious and burdensome symptoms like chronic pain and headaches, weakness, the need for psychiatric support, and issues with mobility, vision, or hearing. Cosmetic appearance/tumor visibility were mentioned by participants who indicated that NF1-PN has a moderate or low impact on their lifestyle.

The need for frequent appointments, monitoring, and tests was noted by the majority of participants in this study and may add to the overall disease burden experienced by patients and their caregivers. This finding is supported by a recent retrospective cohort study, which indicated that patients with NF1 demonstrate increased hospital utilization compared with matched controls.^35^ These findings suggest that involvement of a multidisciplinary team, as well as patient support or care coordinators, may help to ease the healthcare burden for adults, and may support delivery of comprehensive care.

The need for education and guidance when transitioning from pediatric care to adult healthcare management of NF1-PN has previously been noted as an unmet need,^36^ and was further described in a recently published paper by Bischof et al. (2025).^37^ While the majority of adult patients and caregivers participating in this study reported that their transition from pediatric to adult care was smooth, a review conducted in the United States between 2021 and 2022 reported that only 18% of all children aged 12-18 years received the services needed for the transition.^37^^,^^38^ Furthermore, Bischoff et al. (2025) recommended providing assistance with insurance issues, support from a care coordinator or social worker, and appointment reminders from HCPs during care transition. In our analysis, the transition to adult care was typically driven by the pediatric care team and/or insurance. Aging out of pediatric practice was commonly cited as the reason for transitioning to an adult care team; insurance coverage changes were also noted as a reason to transition to an adult care team. Most recalled the transition to adult care was made straightforward by the provision of recommendations and referrals by the pediatric care team, though some patients/caregivers struggled during the transition process. Encouragingly, although not all participants in this study received the level of care recommended by Bischoff et al. (2025),^37^ lapses in care while patients were transitioning to adult HCPs were infrequent. When lapses did occur, it was related to factors such as scheduling barriers, location of expert HCPs (no NF1 clinic nearby), financial insecurity, and lack of insurance; as well as the perception of a lack of treatments or cures available for NF1-PN.

Consistent with the findings of a qualitative survey study by Rietman et al. (2018) involving adults with NF1 and parents of adults with NF1 in the Netherlands,^20^ our study identified several unmet needs when transitioning to adult care. In instances where their pediatric provider was unable to provide a referral, participants reported difficulty in finding an appropriate HCP to manage and treat NF1-PN.

The patient and caregiver responses reflected the need for treatments for NF1-PN in adult patients. At the time of the study, there were no treatments approved for adults and none of the respondents in this study reported receiving MEK inhibitors. Respondents identified the desire for treatments that would slow or stop tumor growth, reduce the size of PN, improve pain management, have minimal side effects, and be covered by insurance.

One of the strengths of this study was the use of a quota approach, which meant a broad picture of patient experiences could be gathered; this was missing in US settings previously. A qualitative design was used to develop insight and direction rather than quantitatively projectable measures. However, due to the small sample size, the recruitment methods used, and the research objectives themselves, this study was limited to being exploratory in nature. Although some findings were consistent across adult patients with NF1-PN and caregivers of adult patients with NF1-PN, the heterogenous nature of the disease resulted in considerable data variability.

The small sample size may have limited the opportunity to identify differences in perspectives and challenges facing different patient groups based on age or the time of transition to adult care. Saturation thresholds can vary dramatically between different qualitative research designs.^32^ A saturation approach was not used in this study due to the rare nature of NF1-PN; however, in-line with the literature a threshold of 12-20 participants was deemed sufficient to provide directional insights to identify themes and answer research questions.^32^ The authors feel that the number and quality of the responses provided in this study are sufficient to evidence the themes that were assessed. Disease severity was not captured and, therefore, not included in the analysis. Disease characteristics such as PN location and size vary considerably among patients with NF1-PN and can affect health-related quality of life. PN characteristics, such as location and size, were not captured in this study; expanding on the disease characteristics that were recorded could provide more detailed insights into disease burden. A specific quota for caregivers was not imposed, and due to difficulties with recruitment, the inclusion of only 2 caregivers limits the generalizability of our findings to the wider population caring for adults with NF1. Furthermore, patients and caregivers self-selected, which could have introduced some bias into the study findings. A study assessing a larger sample size, particularly of caregivers, and covering more regions of the United States would be advantageous. Finally, the recruitment process may have introduced some bias as participants with good levels of engagement with their care teams and the healthcare system were selected, which could explain why the majority of included participants reported a smooth transition from pediatric to adult care. Despite these limitations, the findings regarding disease burden and unmet clinical need of adult patients with NF1-PN identified in this study are generally consistent with previously published literature.^11^^,^^20^^,^^33^^,^^34^ Future studies that include a larger population of adults with NF1-PN are needed to enable a more detailed examination of the experience of patients who have not transitioned from pediatric to adult care or who are not currently in care. While participants were asked about symptoms caused by/related to PN, it is possible that they may not have known whether signs and symptoms, such as chronic headaches or vision impairment, were PN related or were manifestations of NF1 that were independent of their PN. PN can lead to complications, including disfigurement, functional impairment, and compression of adjacent organs, therefore it can be difficult to determine the origin of individual symptoms.^9^^,^^15^^,^^39^ Further studies could aim to differentiate NF1- and PN-related terms in more depth to understand these signs and symptoms better.

In conclusion, the findings from this study indicate that NF1-PN has a profound impact on the lives of adult patients despite variability in symptoms. Patients and caregivers identified several unmet needs in the management of NF1-PN, including the need for improved PN treatments and for additional education regarding PN and potential treatment modalities. Patients had varied experiences transitioning from pediatric to adult care, highlighting an additional unmet need in the management of NF1-PN. This study highlights important gaps in disease management and adds to existing literature on the burden of NF1-PN in adults by providing a direct account from the perspective of patients and caregivers.

## Supplementary Material

vdag033_Supplementary_Data

## Data Availability

The data collected and analyzed during this survey-based study are not publicly available due to participant confidentiality and restrictions imposed by data protection regulations (including GDPR, HIPAA, PIPEDA, PIPL, APPI, or other relevant laws). De-identified and aggregated data may be available from the corresponding author or research sponsor upon reasonable request, subject to review and approval in accordance with applicable privacy and ethical guidelines. Any shared data will exclude personally identifiable information to protect participant privacy.
