# Supplementary material for: Adult patient and caregiver perspectives on the impact of NF1-PN: Insights from a US qualitative survey
Source: Neurooncol Adv. 2026 Feb 16;8(1):vdag033. doi: 10.1093/noajnl/vdag033 (PMC12990978; doi:10.1093/noajnl/vdag033)
Supplement: vdag033_Supplementary_Data [file vdag033_supplementary_data.docx]

Adult Patient and Caregiver Perspectives on the Impact of NF1-PN: Insights from a US Qualitative Survey

Phioanh L. Nghiemphu,^1^ Conrad L. Cordova,^2^ Alyssa Bowling,^3^ Abby Crites,^4^
Xiaoqin Yang,^5^ and Theresa Dettling^6^

## Author affiliations

^1^Department of Neurology, David Geffen School of Medicine, University of California, Los Angeles, CA, USA.

^2^Patient author.

^3^Global Medical Affairs, Alexion, AstraZeneca Rare Disease, Boston, MA, USA.

^4^Advisory & Brand Intelligence, IQVIA, Durham, NC, USA.

^5^Value and Implementation Outcomes Research, Merck & Co., Inc., Rahway, NJ, USA.

^6^US Health Economics and Outcomes Research, Alexion, AstraZeneca Rare Disease, Boston, MA, USA.

Running title

Perspectives of US adult patients with NF1-PN

Corresponding author

Theresa Dettling,

Alexion, AstraZeneca Rare Disease,

121 Seaport Blvd, Boston, MA 02210, USA

Phone number: +1 828 302 8521

Email: [Terry.Dettling@alexion.com](mailto:Terry.Dettling@alexion.com)

Supplementary Material

Supplementary Methods S1. Caregiver discussion guide

| **Section 1: Introduction (5 minutes)** |
| --- |

1. Introduction and Orientation
   1. Hello, my name is <>. I’d like to thank you for agreeing to participate in today’s interview. I am conducting this study for IQVIA, an independent healthcare marketing company.
   2. Information collected during this discussion is for market research purposes only; it is in no way intended to be promotional. This market research has been funded and supported by a pharmaceutical company.
   3. Everything said is held in confidence, and no one will call you as a result of this discussion for any marketing purposes. Moderator has no vested interest in outcome of research; please be open and candid with views.
   4. Colleagues and clients may be listening on a muted line, audio-taped for later review; only research team will have access to the recorded materials and are bound by same Codes of Conduct and Confidentiality as moderator.
   5. Purpose: You had mentioned that you care for someone who was diagnosed with NF1-PN. Today we will be talking to you about their experiences with **Neurofibromatosis type 1 with Plexiform Neurofibromas (NF1-PN)**.
   6. Interview lasts up to 45-minutes; participation strictly voluntary at all stages of interview process.
   7. We are asked to pass on to our client details of adverse events or product quality complaints that you may disclose to us during the course of our discussion -- even if you have already reported it directly to the company or the regulatory authorities.

***[If respondent asks for more information:*** An adverse event is any untoward medical occurrence in a patient and may include any unfavorable and unintended symptom, sign, disease, condition or test abnormality, whether or not it is considered related to the product.***]***

In order for our Client to comply with legal and regulatory requirements, we may need to disclose to our Client your identity and the information you provided related to the adverse event or product quality complaints. The disclosed information will be used by our Client solely for the purpose of complying with legal and regulatory requirements.

With the exception of the adverse events reporting requirements, your identity, and any information you provide in the survey will be treated as confidential. Do you understand and accept those terms and conditions and are you willing to proceed with the research on those terms? **[If Respondent says YES, proceed with interview]**

| **Section 2: Background and Diagnosis (10 minutes)** |
| --- |

1. Tell me a little bit about yourself: age, family, hobbies
2. How long ago was the person you care for diagnosed with NF1-PN?
   1. Were they diagnosed with NF1 **without** plexiform neurofibromas first?
      1. **If Yes:** How long after their diagnosis of NF1 without PNs were they formally diagnosed with PNs?
3. **If Dxed with NF1 without PNs first:** What were their first symptoms of NF1?
   1. How old were they when diagnosed with NF1?
   2. Do you recall how long it took to reach a formal diagnosis of NF1?
   3. What was the process to get the NF1 diagnosis (e.g., number of doctor visits, tests, etc.)?
      1. Were they misdiagnosed with any other condition(s) before they found out it was NF1? If so, what condition(s)?
   4. What doctor(s) were involved in the NF1 diagnosis (i.e., specialties)?
      1. How did you or the person you care for find the doctor(s) that diagnosed them? Were the doctor(s) referred to you or the person you care for?
   5. Prior to receiving an NF1 diagnosis, was the person you care for prescribed any medication(s) for the symptoms they were experiencing?
   6. What role did you play in the medical care of the person you care for during the time of their NF1 diagnosis?
4. What were their first symptoms of NF1-PN?
   1. **If Dxed with NF1 without PNs first:** How did symptoms differ when they were diagnosed with NF1 vs NF1-PN, if at all?
   2. How old were they when diagnosed with NF1-PN?
   3. Do you recall how long it took to reach a formal diagnosis of NF1-PN?
   4. What was the process to get diagnosed (e.g., number of doctor visits, tests, etc.)?
      1. Were they misdiagnosed with any other condition(s) before they found out it was NF1-PN? If so, what condition(s)?
   5. What doctor(s) were involved in their NF1-PN diagnosis (i.e., specialties)?
      1. How did you or the person you care for find the doctor(s) that diagnosed them? Were they referred to you or the person you care for?
      2. **If Dxed with NF1 without PNs first:** Did the doctor(s) differ from those who were involved in their NF1 diagnosis?
   6. Prior to receiving an NF- PN diagnosis, was the patient you care for prescribed any medication(s) for the symptoms they were experiencing?
   7. What role did you play in the medical care of the person you care for during the time of their NF1-PN diagnosis?
      1. Are you involved with making decisions about medical care currently?
      2. Do you support this person in any other ways that make it easier for them to manage their NF1-PN?
5. Has this person been diagnosed with any other conditions associated with NF1?
   - 1. **If Yes:** Are they receiving any treatment or taking medications for other conditions?
6. Tell me about their experiences living with NF1-PN since diagnosis. How has the condition impacted their lifestyle?
   1. What are challenges they experience, if any, in their day-to-day life?
   2. What changes have they made in their day-to-day life since diagnosis, if any?
7. What role do you play in helping the person you care for manage living with NF1-PN? What do you think are the most helpful aspects of care that you provide?

| **Section 3: Physician Dynamics and Transition of Care (10 minutes)** |
| --- |

1. When was the last time they saw a doctor regarding their NF1-PN?
   1. What is the typical amount of time between office visits?
      1. Do you or the patient you care for ever request appointments outside of regularly scheduled appointments? If so, how often and what are the reasons for requesting those appointments?
   2. Has there ever been a time where they’ve gone longer than what is ‘typical’ between office visits? How long?
      1. Was this recommended by your doctor(s)?
         1. **If Yes:** Do you know why?
         2. **If No:** What prevented the person you care for from visiting their doctor(s) more frequently?
      2. What made them (or would make them) return to see a doctor regarding their NF1-PN after the extended break between office visits? **Probe:** symptoms, encouragement from family, etc.
2. What doctor(s) do they see to help them manage their NF1-PN? Do they receive care from one doctor or a team of doctors?
   1. **If Team:**
      1. How many doctors are involved?
      2. Who is involved? What are their specialties?
      3. What is each of their roles in managing your NF1-PN?
         1. Which doctor(s) help manage the symptoms of NF1?
         2. Which doctor(s) help manage the plexiform neurofibromas?
      4. Do they meet with some of them more frequently than others? If so, who and why?
      5. Where does the patient you care for go to see their doctors? **Probe:** Hospital, office
   2. **If one doctor:** What is their specialty?
      1. Where does the patient you care for go to see their doctors? **Probe:** Hospital, office
      2. Does this physician help manage symptoms of both NF1 and the plexiform neurofibromas?
   3. How far do they have to travel to see their doctor(s)? Is that reasonable for them or a challenge?
   4. Were they referred to this physician(s)?
      1. **If Yes:** By whom?
      2. **If No:** How did you or the patient you care for find the physician(s)?
   5. How long have they seen this doctor(s) to manage their NF1-PN?
      1. How often, if ever, have they changed the doctor(s) that manage their NF1-PN?
         1. **If changed:** What prompted the change?
   6. Overall, on a scale of 1 to 5 where 1= not at all satisfied and 5 = extremely satisfied, how satisfied is the person you care for with the doctor(s) they see to manage their NF1-PN? Please explain the rating.
3. **If Dxed as Child (<18yrs):** You mentioned previously that the person you care for was diagnosed with NF1-PN in childhood. Do they see the same doctor(s) that they saw as a child, or do they see different doctor(s) now?
   1. **If different:**
      1. How old were they when they transitioned from a pediatric doctor to an adult doctor to manage their NF- PN?
      2. Who initiated the transition? Did the initiate or the doctor(s)?
      3. How involved were their pediatric doctor(s) in the transition process? Did they refer the adult practitioner?
      4. What was the transition process like? Was it easy or difficult for them? What challenges, if any, did they encounter when transitioning from the pediatric to adult doctor(s)?
   2. **If not different:**
      1. Has there been any discussion with their doctor(s) about changing to another doctor since they became an adult? If so, what was discussed?
         1. Has the frequency of doctor visits changed from when they were a child to now as an adult? How so and why?
   3. **If not mentioned previously:** How has your role in caring for this NF1-PN patient changed over time, if at all?
   4. **If Dxed as a Child:** How has your involvement changed during their transition from childhood into adulthood?

| **Section 4: NF1 PN Management (15 minutes)** |
| --- |

1. Can you describe for me what takes place during a typical visit with their doctor(s)?
2. What is discussed in a typical visit? **Probe:** symptoms, medications, QoL
3. Does their doctor tend to ask about specific symptoms at each office visit?
   - 1. **If Yes:** Which one(s)?
4. **If Dxed as Child (<18yrs):** Are any of the symptoms that the person you care for experienced as an adult different from what they experienced as a child?
   - 1. **If Yes:** What is different?
5. Are there any tests that take place regularly to monitor their NF1-PN?
6. How often are plexiform neurofibromas (PNs) monitored, if at all?
7. What is the current management plan for their NF1-PN? Are they currently taking any medication(s) to manage their NF1-PN? **Probe:** Pain management, surgery, other therapy or interventions, etc.
8. How were these medication(s) and/or therapies selected?
9. How did their doctor(s) describe the medication(s) or therapy to you or the person you care for?
10. To what degree did you and/or the person you care for play a role in selecting their management plan?
11. Were any tests needed prior to going on the medication(s)?
12. How long have they been on each medication and/or therapy?
13. How helpful has each medication and/or therapy been in managing their
    NF1-PN?
14. **If taking another MEK Inhibitor (Koselugo/selumetinib is one option), ask:** You mentioned they are taking a MEK inhibitor. Can you tell me the name and how that was prescribed? Part of a clinical trial?
15. **If Koselugo (selumetinib) is mentioned as current Tx, please confirm if treatment initiation was in childhood or adulthood**
16. **If patient has had surgery:** When did they have surgery? How were you and them involved in the decision to have surgery? What was the outcome of surgery?
17. What medication(s) and/or therapies have they used previously, if any?
18. How were these medication(s) and/or therapies selected?
19. Why did they stop using these medication(s) and/or therapies?

**If Koselugo (selumetinib) is mentioned as previous Tx, please confirm if treatment initiation was in childhood or adulthood**

1. How have costs and/or insurance coverage played a role in the medication(s) and/or therapies they use to manage their NF1-PN, if at all?
2. Have costs or insurance coverage made it difficult to get certain NF1-PN medications and/or therapies? How so and which medication(s) and/or therapies? **Probe:** Has it delayed them being able to get medications on time?
3. Have costs or insurance coverage prevented them from getting certain medications and/or therapies altogether? How so and which medication(s) and/or therapies?
4. Have they received any financial support or assistance for their NF1-PN treatment?
5. **If Yes:** What types of financial support or assistance? How did you and/or the patient you care for learn about those resources?
6. **If Dxed as Child:** Are you aware of any medication and/or therapy costs and/or insurance coverage issues that they encountered in childhood that they haven’t experienced as an adult?
   1. **If Yes:** What types of issues did they experience only as a child patient?
   2. Alternatively, have they experienced issues with NF1-PN medication and/or therapy costs and/or insurance coverage as an adult that they didn’t experience in childhood?
      1. **If Yes:** What types of issues have they experienced only as an adult patient?
7. Are you or the patient you care for familiar with any patient support programs for
   NF1-PN patients (i.e., pharmaceutical programs, advocacy organizations, etc.)?
8. **If Yes**:
9. How did you or the patient you care for learn about the program(s)?
10. What aspects of the program(s) are you or the patient you care for familiar with?
11. Do they use them? How have they helped them, if at all?
12. Using the 1 to 5 scale, where 1 = not at all impactful and 5 = extremely impactful, how has NF1-PN impacted…
13. Their ability to participate in school / work? Please explain.
    - 1. What is their current employment status? If working, how has it impacted their employment, if at all?
      2. What is the highest level of education they have completed?
14. Their personal relationships with family and friends? Please explain.
15. Their mental health? Please explain.
16. Their overall quality of life? Please explain.

| **Section 5: Unmet Needs and Final Questions (5 minutes)** |
| --- |

1. If the person you care for could change anything about how their NF1-PN is managed, what would it be and why? **Probe:** Is there anything that you would change about how their NF1-PN is managed? What would it be and why?
2. Overall, if a new NF1-PN medication became available, what would the patient you care for want the new medication to do to make their NF1-PN more manageable for them?
3. What sources of information do you and/or the patient you care for use to learn about NF1-PN, NF1-PN medications and/or therapies?
   1. How often do you use each source of information? Do you keep up with information on NF1-PN or do you tend to only look up information when you have a question or issue?
   2. How did you find out about each source?
   3. What types of information do you look for?
4. Does their doctor talk to you and/or the patient you care for about new NF1-PN medications that are in development, or clinical trials for NF1-PN medications?
5. Is the person you care for involved with any patient advocacy groups? **If Yes:** How did you or the patient you care for hear about them? How have they used them?
6. **Check for any final questions from IQVIA and the client**
7. Thank the respondent and end the interview

Supplementary Methods S2. Patient discussion guide

| **Section 1: Introduction (5 minutes)** |
| --- |

1. Introduction and Orientation
   1. Hello, my name is <>. I’d like to thank you for agreeing to participate in today’s interview. I am conducting this study for IQVIA, an independent healthcare marketing company.
   2. Information collected during this discussion is for market research purposes only; it is in no way intended to be promotional. This market research has been funded and supported by a pharmaceutical company.
   3. Everything said is held in confidence, and no one will call you as a result of this discussion for any marketing purposes. Moderator has no vested interest in outcome of research; please be open and candid with views.
   4. Colleagues and clients may be listening on a muted line, audio-taped for later review; only research team will have access to the recorded materials and are bound by same Codes of Conduct and Confidentiality as moderator.
   5. Purpose: Today we will be talking to you about your experiences with **Neurofibromatosis type 1 with Plexiform Neurofibromas (NF1-PN)**.
   6. Interview lasts up to 45-minutes; participation strictly voluntary at all stages of interview process.
   7. We are asked to pass on to our client details of adverse events or product quality complaints that you may disclose to us during the course of our discussion -- even if you have already reported it directly to the company or the regulatory authorities.

***[If respondent asks for more information:*** An adverse event is any untoward medical occurrence in a patient and may include any unfavorable and unintended symptom, sign, disease, condition or test abnormality, whether or not it is considered related to the product.***]***

In order for our Client to comply with legal and regulatory requirements, we may need to disclose to our Client your identity and the information you provided related to the adverse event or product quality complaints. The disclosed information will be used by our Client solely for the purpose of complying with legal and regulatory requirements.

With the exception of the adverse events reporting requirements, your identity, and any information you provide in the survey will be treated as confidential. Do you understand and accept those terms and conditions and are you willing to proceed with the research on those terms? **[If Respondent says YES, proceed with interview]**

| **Section 2: Background and Diagnosis (10 minutes)** |
| --- |

1. Tell me a little bit about yourself: age, family, hobbies
2. How long ago were you diagnosed with NF1-PN?
   1. Were you diagnosed with NF1 **without** plexiform neurofibromas first?
      1. **If Yes:** How long after your diagnosis of NF1 without PNs were you formally diagnosed with PNs?
3. **If Dxed with NF1 without PNs first:** What were your first symptoms of NF1?
   1. How old were you when diagnosed with NF1?
   2. Do you recall how long it took to reach a formal diagnosis of NF1?
   3. What was the process to get the NF1 diagnosis (e.g., number of doctor visits, tests, etc.)?
      1. Were you misdiagnosed with any other condition(s) before you found out it was NF1? If so, what condition(s)?
   4. What doctor(s) were involved in the NF1 diagnosis (i.e., specialties)?
      1. How did you find the doctor(s) that diagnosed you? Were they referred to you?
   5. Prior to receiving an NF1 diagnosis, were you prescribed any medication(s) for the symptoms you were experiencing?
   6. Was there anyone else in your life (a family member, friend, caregiver) that was involved in your medical care at the time of your NF1 diagnosis? What role did they play?
4. What were your first symptoms of NF1-PN?
   1. **If Dxed with NF1 without PNs first:** How did symptoms differ when you were diagnosed with NF1 vs NF1-PN, if at all?
   2. How old were you when diagnosed with NF1-PN?
   3. Do you recall how long it took to reach a formal diagnosis of NF1-PN?
   4. What was the process to get diagnosed (e.g., number of doctor visits, tests, etc.)?
      1. Were you misdiagnosed with any other condition(s) before you found out it was NF1-PN? If so, what condition(s)?
   5. What doctor(s) were involved in your NF1-PN diagnosis (i.e., specialties)?
      1. How did you find the doctor(s) that diagnosed you? Were they referred to you?
      2. **If Dxed with NF1 without PNs first:** Did the doctor(s) differ from those who were involved in your NF1 diagnosis?
   6. Prior to receiving an NF1-PN diagnosis, were you prescribed any medication(s) for the symptoms you were experiencing?
   7. Was there anyone else in your life (a family member, friend, caregiver) that was involved in your medical care at the time of your NF1-PN diagnosis? What role did they play?
      1. Are they involved with making decisions about your medical care currently?
      2. Do they support you in any other ways that make it easier to manage your NF1-PN?
5. Have you been diagnosed with any other conditions associated with NF1?
   1. **If Yes:** Are you receiving any treatment or taking medications for other conditions?
6. Tell me about your experiences living with NF1-PN since diagnosis. How has the condition impacted your lifestyle?
   1. What are challenges you experience, if any, in your day-to-day life?
   2. What changes did you make in your day-to-day life since diagnosis, if any?

| **Section 3: Physician Dynamics and Transition of Care (10 minutes)** |
| --- |

1. When was the last time you saw a doctor regarding your NF1-PN?
2. What is the typical amount of time between office visits?
   - 1. Do you ever request appointments outside of regularly scheduled appointments? If so, how often and what are the reasons you’ve requested those appointments?
3. Has there ever been a time where you’ve gone longer than what is ‘typical’ between office visits? How long?
   - 1. Was this recommended by your doctor(s)?
        1. **If Yes:** Do you know why?
        2. **If No:** What prevented you from visiting your doctor(s) more frequently?
     2. What made you (or would make you) return to see a doctor regarding your NF1-PN after the extended break between office visits? **Probe:** symptoms, encouragement from family, etc.
4. What doctor(s) do you see to help you manage your NF1-PN? Do you receive care from one doctor or a team of doctors?
5. **If Team:**
   - 1. How many doctors are involved?
     2. Who is involved? What are their specialties?
     3. What is each of their roles in managing your NF1-PN?
        1. Which doctor(s) help you manage symptoms of NF1?
        2. Which doctor(s) help you manage the plexiform neurofibromas?
     4. Do you meet with some of them more frequently than others? If so, who and why?
     5. Where do you go to see your doctors? **Probe:** Hospital, office
6. **If one doctor:** What is their specialty?
   - 1. Where do you go to see your doctors? **Probe:** Hospital, office
     2. Does this physician help you manage symptoms of both NF1 and the plexiform neurofibromas?
7. How far do you have to travel to see your doctor(s)? Is that reasonable for you or a challenge?
8. Were you referred to this physician(s)?
   - 1. **If Yes:** By whom?
     2. **If No:** How did you find the physician(s)?
9. How long have you seen this doctor(s) to manage your NF1-PN?
   - 1. How often, if ever, have you changed the doctor(s) that manage your NF1-PN? **If changed:** What prompted the change?
10. Overall, on a scale of 1 to 5 where 1= not at all satisfied and 5 = extremely satisfied, how satisfied are you with the doctor(s) you see to manage your
    NF1-PN? Please explain your rating.
11. **If Dxed as Child (<18yrs):** You mentioned previously that you were diagnosed with
    NF1-PN in childhood. Do you see the same doctor(s) that you saw as a child, or do you see different doctor(s) now?
12. **If different:**
    - 1. How old were you when you transitioned from a pediatric doctor to an adult doctor to manage your NF1-PN?
      2. Who initiated the transition? Did you initiate or the doctor(s)?
      3. How involved were your pediatric doctor(s) in the transition process? Did they refer the adult practitioner?
      4. What was the transition process like? Was it easy or difficult for you? What challenges, if any, did you encounter when transitioning from the pediatric to adult doctor(s)?
13. **If not different:**
    - 1. Has there been any discussion with your doctor(s) about changing to another doctor since you became an adult? If so, what was discussed?
      2. Has the frequency of doctor visits changed from when you were a child to now as an adult? How so and why?
14. **If not mentioned previously:** Regarding the person involved in your care, how has their role changed over time, if at all?
15. **If Dxed as a Child:** Has their involvement changed during your transition from childhood into adulthood?

| **Section 4: NF1 PN Management (15 minutes)** |
| --- |

1. Can you describe for me what takes place during a typical visit with your doctor(s)?
2. What do you discuss in a typical visit? **Probe:** symptoms, medications, QoL
3. Does your doctor tend to ask about specific symptoms at each office visit?
   - 1. **If Yes:** Which one(s)?
4. **If Dxed as Child (<18yrs):** Are any of the symptoms that you experience as an adult different from what you experienced as a child?
   - 1. **If Yes:** What is different?
5. Are there any tests that take place regularly to monitor your NF1-PN?
6. How often are plexiform neurofibromas (PNs) monitored, if at all?
7. What is your current management plan for your NF1-PN? Are you currently taking any medication(s) to manage your NF1-PN? **Probe:** Pain management, surgery, other therapy or interventions, etc.
8. How were these medication(s) and/or therapies selected?
   - 1. How did your doctor(s) describe the medication(s) or therapy to you?
     2. To what degree did you play a role in selecting your management plan?
     3. Were any tests needed prior to going on the medication(s)?
9. How long have you been on each medication and/or therapy?
10. How helpful has each medication and/or therapy been in managing your
    NF1-PN?
11. **If taking a MEK Inhibitor (Koselugo/selumetinib is one option), ask:** You mentioned you’re taking a MEK inhibitor. Can you tell me the name and how it was prescribed to you? Part of a clinical trial?

**If Koselugo (selumetinib) is mentioned as current Tx, please confirm if treatment initiation was in childhood or adulthood**

1. **If patient has had surgery:** When did you have surgery? How were you and your caregiver involved in the decision to have surgery? What was the outcome of surgery?
2. What medication(s) and/or therapies have you used previously, if any?
3. How were these medication(s) and/or therapies selected?
4. Why did you stop using these medication(s) and/or therapies?

**If Koselugo (selumetinib) is mentioned as previous Tx, please confirm if treatment initiation was in childhood or adulthood**

1. How have costs and/or insurance coverage played a role in the medication(s) and/or therapies you use to manage your NF1-PN, if at all?
2. Have costs or insurance coverage made it difficult to get certain NF1-PN medications and/or therapies? How so and which medication(s) and/or therapies? **Probe:** Has it delayed you being able to get medications on time?
3. Have costs or insurance coverage prevented you from getting certain medications and/or therapies altogether? How so and which medication(s) and/or therapies?
4. Have you received any financial support or assistance for your NF1-PN treatment?
5. **If Yes:** What types of financial support or assistance? How did you learn about those resources?
6. **If Dxed as Child:** Are you aware of any medication and/or therapy costs and/or insurance coverage issues that you encountered in childhood that you haven’t experienced as an adult?
   1. **If Yes:** What types of issues did you experience only as a child patient?
   2. Alternatively, have you experienced issues with NF1-PN medication and/or therapy costs and/or insurance coverage as an adult that you didn’t experience in childhood?
      1. **If Yes:** What types of issues have you experienced only as an adult patient?
7. Are you familiar with any patient support programs for NF1-PN patients (i.e., pharmaceutical programs, advocacy organizations, etc.)?
8. **If Yes**:
   - 1. How did you learn about the program(s)?
     2. What aspects of the program(s) are you familiar with?
     3. Do you use them? How have they helped you, if at all?
9. Using the 1 to 5 scale, where 1 = not at all impactful and 5 = extremely impactful, how has NF1-PN impacted…
10. Your ability to participate in school / work? Please explain.
    - 1. What is your current employment status? If working, how has it impacted your employment, if at all?
      2. What is the highest level of education you have completed?
11. Your personal relationships with family and friends? Please explain.
12. Your mental health? Please explain.
13. Your overall quality of life? Please explain.
14. Do you have a friend, family member, or someone else involved in helping you manage living with NF1-PN?
15. **If yes:** What role does he/she play? **Probe:** Is he/she involved in selecting or managing your medication(s) and/or therapies? What are the most helpful aspects of care this person(s) provides?

| **Section 5: Unmet Needs and Final Questions (5 minutes)** |
| --- |

1. If you could change anything about how your NF1-PN is managed, what would it be and why?
2. Overall, if a new NF1-PN medication became available, what would you want the new medication to do to make your NF1-PN more manageable for you?
3. What sources of information do you use to learn about NF1-PN, NF1-PN medications and/or therapies?
   1. How often do you use each source of information? Do you keep up with information on NF1-PN or do you tend to only look up information when you have a question or issue?
   2. How did you find out about each source?
4. What types of information do you look for? Does your doctor talk to you about new
   NF1-PN medications that are in development, or clinical trials for NF1-PN medications?
5. Are you involved with any patient advocacy groups?
   1. **If Yes:** How did you hear about them? How have you used them?
6. **Check for any final questions from IQVIA and the client**
7. Thank the respondent and end the interview

Supplementary Table 1. Patient and caregiver responses when asked what the transition process from pediatric to adult care was like

| **Theme** | **Number of participants** | **Relevant quotes** |
| --- | --- | --- |
| Easy/uncomplicated | 5 | “It's been relatively straightforward. It was actually easy” |
|  |  | “They just gave me a list of potential doctors, I did my research and picked one that I thought would be a good fit; there wasn't a whole lot to it” |
|  |  | “My parents were involved in the transition stage when I was younger. I think it was easy” |
|  |  | “It was fine. It was no problem” |
|  |  | “I think the transition was pretty smooth. It was a little nerve-racking at first but once I was in there it was smooth” |
| Experienced challenges finding the right HCP to manage and treat NF1-PN | 3 | “I think it was difficult at first because I was so used to my doctor” |
|  |  | “It took a lot of time trying to get set up with a new doctor and get the appointments going” |
|  |  | “Finding a doctor that I'm comfortable with was difficult and time consuming” |
| Frustration with a different level or lack of care and understanding about NF1 PN symptoms | 1 | “I don't really feel like the team is doing enough and things are being dismissed” |
| Feeling that they were not being heard by their adult care team compared with pediatric care team | 2 | “I refused to go initially because I didn't like the geneticist. It was a personality conflict. She was very pessimistic and almost suggested that NF1 patients should just sit down and give up” |
|  |  | “I just don't think that the team that I'm currently with is taking things as seriously as they should”  “I had to switch last year to the adult practice. Because my team couldn't offer any more. I switched over to a genetic-based team. They're just not really doing much to help, aside from sending me off to pain management” |

**Abbreviations**: NF1 = neurofibromatosis type 1; PN = plexiform neurofibroma.

Lay Summary

People with neurofibromatosis type 1 (NF1) can develop tumors called plexiform neurofibromas (PN). PN are often painful and can have a notable impact on adults with NF1 and their caregivers. This study aimed to improve understanding of disease burden, healthcare experience, and the unmet needs of adults with NF1-PN in the US, based on interviews with both patients and caregivers. Patients reported living with multiple conditions associated with NF1, including pain disorders, psychiatric disorders, and chronic migraines. NF1-PN had a profound impact on daily living, work, school, relationships, mental health, and emotional health. Some patients stopped seeing a doctor as they moved from childhood to adult settings due to factors such as time constraints, physician location, financial insecurity, lack of insurance, and perception of no available treatment/cure. Key unmet needs included a desire to be more informed about their care and improved NF1-PN treatment options; medications that stop or slow PN growth were highly desirable. These findings provide a unique patient and caregiver perspective on the burden experienced and highlight unmet needs.
